# The knowledge, attitudes and practices of doctors, pharmacists and nurses on antimicrobials, antimicrobial resistance and antimicrobial stewardship in South Africa

**DOI:** 10.4102/sajid.v36i1.262

**Published:** 2021-04-21

**Authors:** Reshma Balliram, Wilbert Sibanda, Sabiha Y. Essack

**Affiliations:** 1 Antimicrobial Research Unit, Faculty of Health Sciences, University of KwaZulu-Natal, Durban, South Africa; 2 Department of Public Health, Faculty of Health Sciences, University of KwaZulu-Natal, Durban, South Africa

**Keywords:** antimicrobials, antimicrobial resistance, antimicrobial stewardship, knowledge, attitudes, practices

## Abstract

**Background:**

Sustained injudicious and indiscriminate use of antimicrobials has exerted selection pressure for developing antimicrobial resistance (AMR), requiring behaviour change from healthcare professionals (HCPs) based on their knowledge, attitudes and practices (KAP) on antimicrobials, AMR and antimicrobial stewardship (AMS).

**Methods:**

A cross-sectional online questionnaire-based survey was conducted nationally amongst doctors, pharmacists and nurses from November 2017 to January 2018. The questionnaire comprised demographic information and KAP questions.

**Results:**

Respondents comprised of 1120 doctors, 744 pharmacists and 659 nurses. Antimicrobial resistance was considered a severe problem globally and nationally by majority of HCPs. Self-assessment of knowledge revealed gaps in understanding of antimicrobials, AMR and AMS. Confidence scores in prescribing by doctors, pharmacists and nurses were 57.82%, 32.88% and 45.28%, respectively. Doctors, 441 (45.2%) indicated no confidence in using combination therapy. Prescribing correctly showed a confidence level of 33.99% from 436 doctors, 41.88% from nine pharmacists and 35.23% from 107 nurses. Healthcare professionals (1600 [91.22%]) stated educational campaigns would combat AMR. Only 842 (40.13%) HCPs attended training on these topics and 1712 (81.60%) requesting more education and training.

**Conclusion:**

This is the first comparative survey on KAP of practising doctors, pharmacists and nurses in South Africa. Doctors had the highest knowledge score followed by nurses and pharmacists. Practice scores did not corroborate knowledge and the higher attitude scores. Gaps in KAP were evident. Healthcare professionals indicated the need for more education and training, thus requiring a review of pre-service and in-service education and training in addition to continued professional development programmes for practising HCPs.

## Introduction

The injudicious and irrational use of antimicrobials, *vis-à-vis*, incorrect clinical indication, dosing and administration, and, non-complaince of patients have been implicated in the development of antimicrobial resistance (AMR).^1^ Antimicrobial resistance, an escalating threat globally, is of concern in human and animal health, the food industry and agriculture.^2^ It adversely affects treatment, increases morbidity and mortality, results in extended hospital stays and necessitates more expensive, and often more toxic, treatment options.^3^ According to best available data, an estimated 700 000 people worldwide die of resistant bacterial infections a year, and it is estimated that this may increase to 10 million people dying a year at a cost of 100 trillion USD by 2050.^4^ Change lies in the hands of healthcare professionals (HCPs) responsible for the prescription, dispensing and administration of antimicrobial medicines to patients, namely doctors, pharmacists and nurses, respectively.

The Global Action Plan (GAP) on AMR addresses AMR through five strategic objectives. Of these, strategic objective 1 is increased awareness of AMR through effective communication, education and training.

This would be achieved by each member state implementing interventions to: (1) increase national awareness of AMR via programmes that target the different audiences in human health, animal health and the environment, (2) incorporate AMR as a core component in professional education, training and certification, (3) incorporate antimicrobial use and resistance into the school syllabus to improve understanding and further awareness with all information being accurate and relevant, (4) prioritise AMR as an important health issue requiring urgent action from all governmental departments and (5) create a multisectoral committee to address AMR from a One Health perspective.^2^ Education for all HCPs on antimicrobial prescribing needs to begin at undergraduate levels, and should continue in post-graduation with specific training in using treatment guidelines.^5^ The range of antimicrobial prescribers has been changing, with legislation allowing nurses, pharmacists and emergency care personnel to prescribe antimicrobials under certain conditions in South Africa (SA).^6,7,8^ Healthcare professionals are responsible for managing antimicrobials, namely prescribing, dispensing and administering antimicrobials to patients. They must be knowledgeable and up-to-date on issues related to antimicrobials, AMR and antimicrobial stewardship (AMS). This study therefore ascertained the knowledge, attitudes and practices (KAP) of doctors, pharmacists and nurses in order to identify gaps for educational intervention.

## Methods

### Study design and population

A descriptive cross-sectional online survey was conducted nationally. Study sample included 15 111 (40.77%) pharmacists, including community-service pharmacists registered with the South African Pharmacy Council (SAPC). There were also 16 260 (43.87%) doctors and 5695 (15.36%) nurses, comprising of 5630 registered nurses and 65 enrolled nurses, who subscribed to Medpages, an SA database with contact information of HCPs in SA. The SAPC provided contact information of pharmacists and community-service pharmacists to the principal investigator, which was used to email the survey questionnaires. Medpages distributed the survey questionnaire to all doctors and nurses on their database. The study was undertaken from November 2017 to January 2018.

### Survey instrument

The data were collected using a self-administered, web-based questionnaire with voluntary informed consent.

Questionnaire (Appendix 1) was adapted and piloted from a combination of questionnaires already available in literature.^9,10,11,12^ Responses were anonymous. Questionnaire was divided into four sections and consisted of open-ended, closed-ended (yes or no) and Likert style questions (one of the following options: strongly agree, agree, neutral, disagree, strongly disagree).

The first section collected demographic, academic and professional data of the participants.

Second section consisted of questions that assessed the participants’ knowledge on antimicrobials, AMR and stewardship, contributing factors to AMR, sources of information and confidence in prescribing antimicrobials. Third section evaluated the participants’ attitudes and beliefs on antimicrobials, AMR and their contribution to AMR. Final section was practice-related and involved antimicrobial sensitivity-testing, empiric-prescribing, use of standard treatment guidelines (STGs), advice imparted to patients and prescribing for diagnosed medical conditions.

### Statistical analysis

Dataset was analysed using IBM Statistical Package for the Social Sciences (IBM SPSS) version 25 (IBM Corp. Released 2018. IBM SPSS Statistics for Windows, Version 25.0. Armonk, NY: IBM Corp) using simple descriptive statistics to create frequencies and percentages. Continuous variables, such as ages, were described as mean ± standard deviation or median and compared using the Student’s *t*-test or Wilcoxon test as appropriate. Categorical variables, such as groups, were described as proportions and compared using Chi-square test or Fisher’s exact test as appropriate. The analysis of variance (ANOVA) statistical test was used to compare the KAP scores between the three professions, and the Bonferroni post-hoc test further described the pairwise statistically significant difference between the professions. For each factual question, a mark was allocated when the answer was correct. Other questions were assessed and given a mark for the most appropriate correct response. Responses for ‘strongly agree’ and ‘agree’ were combined as well as for ‘disagree’ and ‘strongly disagree’. The study population had declined from Section 1–4 of the questionnaire, thus completed sections were analysed separately. Partially completed questionnaires were also considered, hence the different ‘*n*’ values per section. The South African STGs were used to assess doctors and nurses’ choices of antimicrobial treatment for given medical conditions and the primary care drug treatment (PCDT) list was used to assess pharmacists’ choice of antimicrobial treatment in Section 4 of the questionnaire.

### Ethical considerations

Ethical approval was obtained from the Humanities and Social Sciences Research Ethics Committee of University of KwaZulu-Natal (HSS/0868/017M) prior to the study.

## Results

Total number of respondents in this study was 2523 (6.81%) HCPs, consisting of 1120 doctors, 744 pharmacists and 659 nurses with response rates of 6.89%, 4.92% and 11.57%, respectively. There were 1520 (60.25%) females and 1003 (39.75%) males. The modal-age groups were 31–40 years (30.71%) for doctors, 21–30 years (30.24%) for pharmacists and 51–60 years (36.87%) for nurses. Amongst nurses, 392 (59.48%) possessed a diploma qualification. A greater proportion of HCPs, that is 1843 (73.05%), occupied jobs in urban areas whilst the number for those in rural areas was 306 (12.13%). Most HCPs, 1205 (47.76%), worked in private practice and 792 (31.39%) worked in public (Appendix 1).

### Knowledge

#### Awareness of antimicrobial resistance

The majority of HCPs (93.37%) perceived AMR to be a serious problem globally (Table 1). Using a one-way ANOVA, there were statistically significant differences in the appreciation of the problem of AMR between the different professions (*p* = 0.002) with nurses being least aware. Similar numbers of HCPs agreed it was a national problem. However, much lower number of HCPs (73.77%) agreed AMR was a serious problem in their hospital or practice and there was a statistically significant difference between them (*p* = 0.011).

**TABLE 1:** Healthcare professionals’ responses on awareness, knowledge and education on antimicrobials, antimicrobial resistance and antimicrobial stewardship.

Variable	Doctors		Pharmacists		Nurses		*p* (ANOVA)
	*n*	%	*n*	%	*n*	%	
**AMR is a serious problem:**							
Globally	940	96.4	594	95.0	445	88.7	0.002
Nationally	942	96.6	599	95.8	402	92.8	-
In your hospital or practice	761	78.1	475	76.0	335	67.2	0.011
Antimicrobials are not effective in treating acute viral infections – True	925	94.9	579	92.6	375	75.3	0.000
Common colds are caused by viruses – True	966	99.1	608	97.3	449	90.2	0.000
Attended workshops and training on either or both AMS and antimicrobials	435	44.6	242	38.7	165	33.1	0.001
Wanted more education and training on AMR, AMS and antimicrobial use	789	80.1	495	79.2	428	85.9	0.000

Note: Nurses: *N* = 498; Pharmacists: *N* = 625; Doctors: *N* = 975.

AMR, antimicrobial resistance; AMS, antimicrobial stewardship.

#### Assessment of knowledge on antimicrobials, antimicrobial resistance and antimicrobial stewardship

**FIGURE 1:**
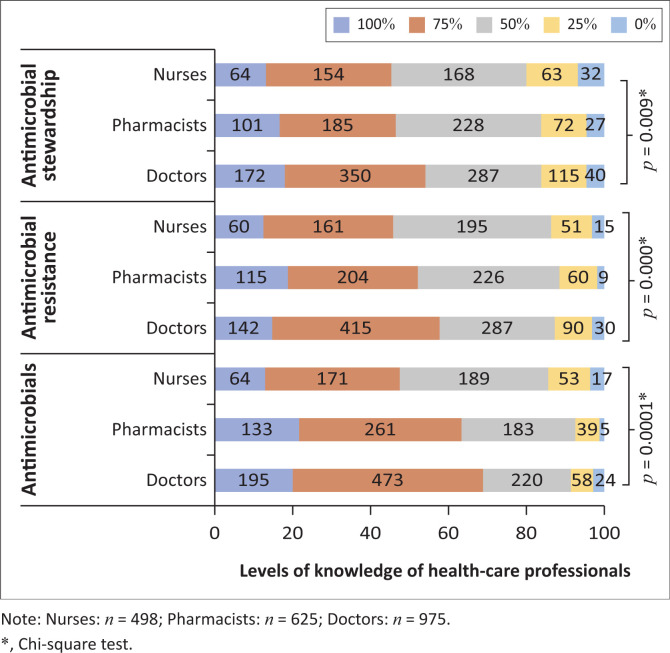
Healthcare professionals’ self-assessment of their levels of confidence on knowledge of antimicrobials, antimicrobial resistance and antimicrobial stewardship.

The self-assessment of knowledge indicated that 791 (37.70%) HCPs were 75% confident of their knowledge on all three topics with 349 (16.6%) HCPs showing 100% confidence (Figure 1). Notably, a greater percentage of nurses (52.40%; 261) had ≤ 50% confidence levels on knowledge of all three topics as compared to pharmacists (45.3%; 283) and doctors (39.3%; 383). Using the ANOVA test, there were statistically significant differences in confidence levels of knowledge on antimicrobials, AMR and AMS between doctors, pharmacists and nurses (*p* = 0.0001, 0.00001 and 0.009, respectively). A Bonferroni post-hoc test indicated that doctors were different from nurses and pharmacists were different from nurses, however doctors and pharmacists were not statistically different.

Varying numbers of HCPs correctly stated antimicrobials were not effective in treating acute viral infections (*p* = 0.000), and a majority of HCPs correctly stated common colds are caused by viruses (Table 1).

#### Contributory factors towards antimicrobial resistance

**FIGURE 2:**
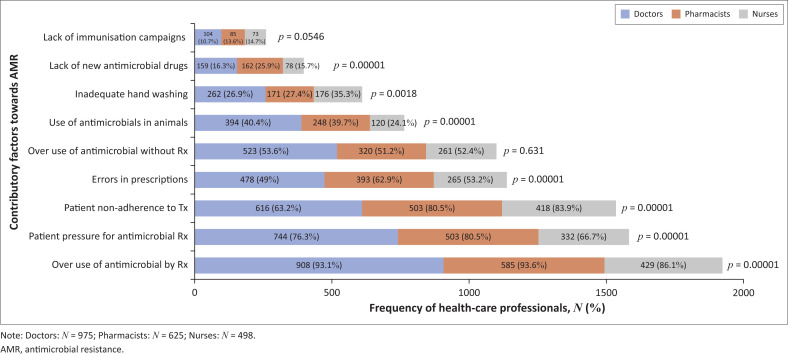
Selection of contributory factors resulting in antimicrobial resistance.

Healthcare professionals identified the overuse of antimicrobials by prescriptions (1922; 91.61%), patient pressure for antimicrobial prescriptions (1579; 75.26%) and non-adherence of patients to prescribed treatment (1537; 73.26%) as most contributory towards AMR, as depicted in Figure 2. The least contributory was the lack of immunisation campaigns (262; 12.49%). Statistically significant differences (*p* < 0.05) were noted between the three groups of HCPs in seven of the nine contributing factors.

#### Confidence in prescribing antimicrobials

Self-assessment on confidence in 10 aspects of prescribing antimicrobials showed 587 (60.21%) doctors were confident in 7 (70%) aspects. No confidence was reported by 433 (44.40%) doctors about using combination therapy when necessary, about when to stop or streamline therapy according to clinical evaluations and investigations and about making decision not to prescribe antimicrobials when there’s fever with no serious criteria (Table 2). A total of 244 (48.93%) nurses stated confidence in six (60%) aspects, and overall 117 (23.50%) nurses stated no confidence in prescribing antimicrobials. Only 206 (33.88%) pharmacists were confident in all aspects of prescribing with 156 (25%) stating no confidence when prescribing antimicrobials. The confidence level of pharmacists and nurses can be gauged from the fact that 221 (35.38%) pharmacists and 116 (23.44%) nurses selected ‘not applicable’ to prescribing antimicrobials. Statistically significant differences (*p* < 0.05) were noted between the three groups of HCPs in eight aspects of prescribing.

**TABLE 2:** Self-assessment on confidence in various aspects of prescribing antimicrobials.

Levels of confidence†	Variable	Confident		Unconfident		*p*-Value (ANOVA)	Statistically significant differences *p* < 0.05 were observed in the following
		*n*	%	*n*	%		
Making an accurate diagnosis of the infection	Doctors	603	61.8	262	35.1	0.000	Between doctors and pharmacists (*p* = 0.00), pharmacists and nurses (*p* = 0.00)
	Pharmacists	154	24.7	166	26.6		
	Nurses	251	50.4	107	21.5		
Decision not to prescribe antimicrobial when patient has fever with no serious criteria and you’re not sure of diagnosis	Doctors	505	51.8	439	45.0	0.000	Between doctors and nurses (*p* = 0.00), pharmacists and nurses (*p* = 0.001)
	Pharmacists	180	28.8	136	21.8		
	Nurses	247	49.6	101	20.3		
Selecting the correct antimicrobial	Doctors	582	59.7	364	37.3	0.024	Between pharmacists and nurses (*p* = 0.023)
	Pharmacists	213	34.1	152	24.3		
	Nurses	231	46.4	108	21.7		
Selecting the correct dosage for the antimicrobial	Doctors	606	62.2	340	34.9	0.005	Between doctors and nurses (*p* = 0.C08)
	Pharmacists	276	44.2	119	19.1		
	Nurses	248	49.8	89	17.9		
Selecting the correct interval for the antimicrobial	Doctors	593	60.8	353	36.2	0.13	Between doctors and nurses (*p* = 0.018)
	Pharmacists	270	43.2	127	20.3		
	Nurses	241	48.4	97	19.5		
Selecting the correct duration for the antimicrobial	Doctors	578	59.3	367	37.6	0.003	Between doctors and nurses (*p* = 0.002)
	Pharmacists	258	41.5	137	21.9		
	Nurses	244	48.8	97	19.5		
Using combination therapy if necessary	Doctors	501	51.3	441	45.2	0.447	No significant differences were observed, *p* > 0.05
	Pharmacists	200	32.0	188	30.1		
	Nurses	192	38.6	146	29.3		
Interpreting microbiological laboratory results	Doctors	589	60.4	354	36.3	0.000	Between doctors and pharmacists (*p* = 0.00), pharmacists and nurses (*p* = 0.003)
	Pharmacists	186	29.8	198	31.7		
	Nurses	220	44.2	142	28.5		
When to stop/streamline the antimicrobial therapy according to clinical evaluations and investigations	Doctors	524	53.7	419	43.0	0.000	Between doctors and pharmacists (*p* = 0.00), pharmacists and nurses (*p* = 0.001)
	Pharmacists	153	24.5	210	33.6		
	Nurses	195	39.2	150	30.1		
Selecting between an intravenous or oral antimicrobial	Doctors	557	57.1	386	39.6	0.000	Between doctors and pharmacists (*p* = 0.00), pharmacists and nurses (*p* = 0.018)
	Pharmacists	165	26.4	190	30.4		
	Nurses	186	37.3	135	27.1		

Note: Nurses: *N* = 498; Pharmacists: *N* = 625; Doctors: *N* = 975.

† , Prescribing antimicrobials represented as a percentage.

ANNOVA, analysis of variance.

The most preferred sources of information on appropriate use of antimicrobials for doctors were STGs (706; 72.5%) followed by South African Medicines Formulary (SAMF) (579; 59.4%). The pharmacists preferred the SAMF (453; 72.5%) and STGs (426; 68.2%), whilst nurses used STGs (343; 68.9%) and indicated equal usage of the SAMF and WHO guidelines (277; 55.6%).

A majority (1972; 93.99%) of HCPs agreed that there were risks associated with the irrational use of antimicrobials, most noted risks being AMR (1729; 82.41%), side effects (675; 32.17%) and adverse drug reactions (486; 23.16%). Only 842 (40.13%) HCPs had attended any workshops or training on antimicrobials and AMS.

A total of 1712 (81.60%) HCPs requested more education and training on antimicrobial use, AMR and AMS.

There was a statistically significant difference in total knowledge of antimicrobials, AMR and AMS between the three groups of HCPs. Using the Bonferroni post-hoc tests, it was observed that doctors scored significantly higher than both the pharmacists and nurses (*p* < 0.05), with the knowledge scores being 65.74%, 60.07% and 60.14%, respectively.

### Attitudes

Doctors (551; 64.1%), pharmacists (354; 68.7%) and nurses (249; 65.5%) disagreed that antimicrobials were safe drugs that could be commonly prescribed. Majority (1689; 96.29%) agreed that prescribing antimicrobials to patients who did not really need them, would ultimately have a negative impact on their health.

#### Strategies to combat antimicrobial resistance

**FIGURE 3:**
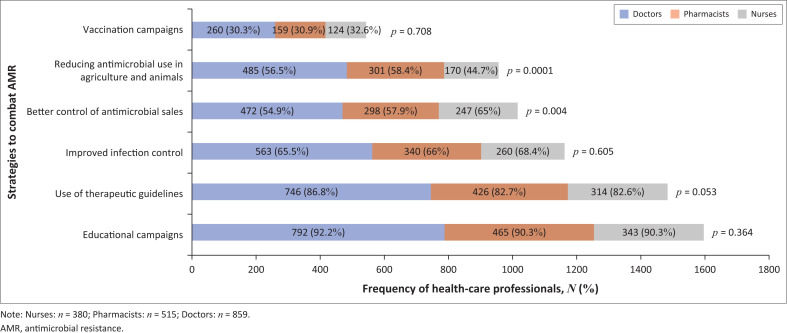
Attitudes on strategies that would combat antimicrobial resistance.

The most important strategies the HCPs believed would aid in combatting AMR were educational campaigns (1600; 91.22%), use of therapeutic guidelines (1486; 84.72%) and improved infection control (1163; 66.31%). Vaccination campaigns (543; 30.96%) were surprisingly reported to be least important. Statistically significant differences (*p* < 0.05) were observed for two strategies (Figure 3).

Varying numbers of HCPs – 600 (69.8%) doctors, 414 (80.4%) pharmacists and 323 (85.0%) nurses – believed skipping or missing a dose or two of antimicrobials contributed to the development of AMR and this difference was statistically significant (*p* = 0.000). A total of 545 (63.4%) doctors, 221 (42.9%) pharmacists and 126 (33.2%) nurses believed they personally contributed towards AMR in some form (*p* = 0.000).

With respect to attitude towards antimicrobials, AMR and AMS, using a one-way ANOVA, there was a statistically significant difference between doctors, pharmacists and nurses with attitude scores of 68.71%, 68.59% and 65.94%, respectively (*p* = 0.013). Using the Bonferroni post-hoc analysis, doctors and pharmacists scored statistically higher than nurses (*p* = 0.014).

### Practices

#### Prescribing antimicrobials

Only 22 (4.43%) pharmacists possessed Section 22A (15) permit, which allowed them to prescribe for PCDT, of which only 16 (3.22%) pharmacists prescribed antimicrobial drugs. Almost a third of nurses (126; 34.00%) possessed the concession permit in terms of Section 22A (12) of the *Medicines and Substances Control Act* of 1965 and Section 38A of the *Nursing Act* of 1978, which allowed nurses to prescribe medicines. However, only 75 (20.22%) prescribed antimicrobial drugs. In this study, 82 (16.50%) pharmacists and 33 (8.89%) nurses prescribed antimicrobials without a licence. Majority of antimicrobials were prescribed by doctors.

#### Antimicrobial stewardship

Six hundred and seventy-eight (80.52%) doctors, 19 (3.82%) pharmacists and 61 (16.44%) nurses prescribed antimicrobials empirically (*p* = 0.000). Four hundred and forty-one (52.38%) doctors, seven (1.41%) pharmacists and 83 (22.37%) nurses sent samples for microbiology testing before initiating antimicrobials. Only 496 (58.90%) doctors, 349 (70.22%) pharmacists and 185 (49.87%) nurses possessed the latest South African STGs (*p* = 0.000). However, only 197 (23.40%) doctors, 42 (8.45%) pharmacists and 66 (17.79%) nurses always used the STGs (*p* = 0.000).

**FIGURE 4:**
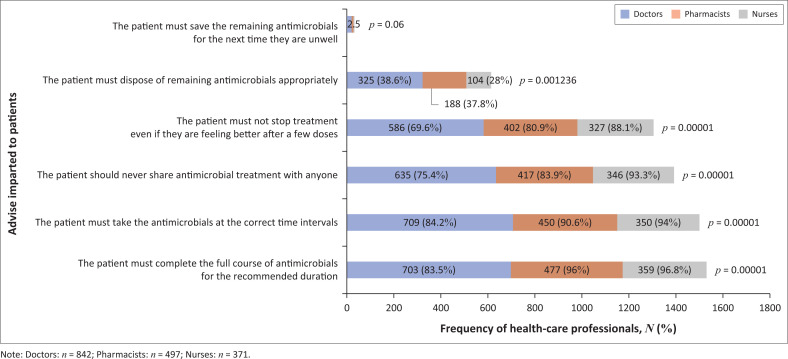
Advice imparted by healthcare professionals to patients on antimicrobial use.

Advice imparted to patients on antimicrobial use is reported in Figure 4. On the safe use of antimicrobials, 309 (83.29%) nurses gave the most correct advice, followed by 404 (81.29%) pharmacists and 630 (74.82%) doctors (*p* < 0.00).

Based on a range of practice scores, between 0 (poor) and 100 (best practice), average scores for doctors, pharmacists and nurses were 57.68% ± 16.42%, 43.14% ± 16.53% and 54% ± 14.34%, respectively. Using an ANOVA test, practice scores for the three groups of HCPs were statistically significantly different (*p* < 0.05).

#### Selection of appropriate antimicrobial treatment

There was a higher percentage of appropriate treatment (41.88%) provided by nine pharmacists compared to 33.99% from 436 doctors and 35.23% from 107 nurses. However, there were fewer pharmacists who were eligible to provide treatment for the given conditions. It must be noted only 1.87% of nurses provided the correct strength, interval and duration for treatment.

## Discussion

To our knowledge, this is the largest national study ascertaining KAP on antimicrobials, AMR and AMS amongst prescribers, dispensers and administrators of antimicrobial medicines to patients. This is the first KAP study amongst doctors, pharmacists and nurses in SA using the same tool and it provides valuable insights and information on strengths and weaknesses of KAP amongst HCPs and indicates areas where interventions are required.

Antimicrobial resistance is an increasingly serious public health threat.^13^ Awareness of AMR is the first step in addressing and reducing this global problem.^2^ We found that majority of HCPs in SA have been sensitised to AMR as a national and global concern, with doctors having most awareness followed by pharmacists and then nurses who had the least conceptual awareness. However, as a group, HCPs’ awareness of AMR was comparable to a study by Burger et al. (2016), in which final year pharmacy students in SA reported 97.7% awareness to AMR as a global problem.^14^ Our HCPs’ national awareness of AMR was similar to the studies by Vaillant et al. (2019), where French HCPs reported 93% national awareness of AMR, and Farley et al. (2018), where 95.8% of primary care prescribers in SA stated AMR was a national problem.^15,16^

The appreciation of the problem of AMR in context of their own work showed there was a much lower awareness of AMR in their hospitals or practices. This being stated, our findings showed higher awareness at practice level compared to the study by Vaillant et al. (2019), who reported 51.5% of French HCPs had awareness of AMR at practice level.^15^ Our research findings with respect to AMR awareness amongst HCPs are similar to Wasserman et al. (2017), who reported that 87% of final year medical students in SA agreed AMR was a global problem and only 61% saw it as a problem in their practice.^17^ Similar results were reported in other studies by Pulcini et al. (2010), where there was 95% national awareness with 63% local awareness for AMR by junior doctors, and Garcia et al. (2011), who reported 97% global and national awareness with 73% local awareness by prescribing doctors.^9,18^ However, our local awareness results contrasted with the study by Salsgiver et al. (2018) where it was reported that majority (89%) of prescribers in the United States agreed AMR was a problem at their hospitals and prescribers actively supported AMS programmes at all hospitals.^19^

Based on their self-assessment, only 16.6% of HCPs were 100% confident in their knowledge of antimicrobials, AMR and AMS. The percentage of HCPs that had 75% confidence in their knowledge of antimicrobials and AMR, respectively, was 43% and 37%. This is in contrast to the report by Dall (2019) whereby 80% of HCPs from 30 European countries stated they possessed sufficient knowledge on correct antimicrobial use and 96% stated having knowledge of AMR.^20^ Several aspects must be comprehensively considered when prescribing antimicrobials and complete confidence in each aspect is essential in achieving positive patient outcomes. In this study, diminished levels of confidence in the 10 aspects of prescribing antimicrobials were observed, with 587 (60.21%) doctors confident in only seven aspects. A third of pharmacists stated confidence in all aspects whilst almost a quarter of the nurses were not confident in prescribing. Wasserman et al. (2017) reported that only a third of the students were confident in prescribing.^17^ Our results are also similar to findings by Pulcini et al. (2010), where it was found junior doctors in France and Scotland shared similar overall confidence (57.61%) in prescribing antimicrobials to that of our doctors (57.82%).^9^

Forty per cent of HCPs had attended or received training on antimicrobials and AMS with majority (81.61%) indicating a need for more education and training. Our research findings are similar to those reported by Burger et al. (2016) whereby only 37.75% of pharmacy students had training on AMS and 90% needed more training.^14^ This was confirmed by Farley et al. (2018) where numerous prescribers requested more education on appropriate antimicrobial use and in Wasserman et al. (2017) where medical students wanted more education to aid in antibiotic prescribing.^16,17^ On the safe use of antimicrobials, close to two-thirds of HCPs indicated an awareness that in general antimicrobials are safe, however continuous use without due regard can have undesired effects. Majority of HCPs indicated that there could be negative effects on patients if antimicrobials were unnecessarily prescribed. The latter finding was supported by Burger et al. (2016).^14^

Numerous contributing factors result in AMR. In this study, the leading causes identified by majority of HCPs were: over-prescription of antimicrobials, patients’ non-adherence to treatment and patient pressure for antimicrobial treatment. Burger et al. (2016) similarly reported widespread and overuse of antimicrobials (94.2%) and poor patient adherence to medication (89.5%) as contributing to AMR.^14^ A similar observation was made by Wasserman et al. (2017), who reported inappropriate use of antimicrobials (> 95%) as the primary cause of AMR.^17^. Similar findings were also reported in other studies by Pulcini et al. (2010) in France and Scotland, where > 90% of the junior doctors selected over-prescription of antimicrobials as being causative; Abera et al. (2014) in Ethiopia, where the nurse prescribers and doctors stated poor patient adherence (86%) and overuse of antimicrobials (80.5%) were responsible for AMR; and Dyar et al. (2014) in Europe, where final year medical students reported over-prescription of antimicrobials (> 95%) resulted in AMR.^9,10,21^

According to our research, the most appropriate resources for antimicrobial selections were STGs, SAMF and international guidelines, respectively. This is in contrast to Wasserman et al. (2017) who reported that medical textbooks (87%), registrars (85%) and consultants (83%) were most common learning resources.^17^ The important strategies chosen for combatting AMR by majority of HCPs were educational campaigns (91.22%) and usage of therapeutic guidelines (84.72%). This finding was similar to Burger et al. (2016) who reported that education on antimicrobial therapy (92.3%), antimicrobial usage policies (86.9%) and development of treatment guidelines (86.2%) would combat AMR.^14^ Interestingly only 60.2% of HCPs were in possession of the latest South African STGs and only 17.8% always used it.

Attitude scores amongst all professions were slightly higher than knowledge scores and practice scores were the lowest. The doctors and pharmacists indicated higher attitude scores than nurses. Doctors obtained the highest knowledge scores. Practice scores did not corroborate the self-proclaimed knowledge and attitude scores.

### Limitations

Gatekeeper permission to contact the HCPs proved problematic. The Protection of Personal Information Act (POPI) precluded access to the databases of the Health Professions Council of SA. The SA Nursing Council and Forum of University Nursing Deans of SA could not assist. The survey was conducted from November 2017 to January 2018, which is a festive period in SA, resulted in reduced participation from professionals.

Participation was voluntary, and, combined with the above constraints; the response rate was relatively poor.

The results should thus be extrapolated to the wider HCP community with caution. Not all the questionnaires were fully completed resulting in different ‘*n*’ values for different sections.

## Conclusion

This is the first comparative survey on KAP of practising doctors, pharmacists and nurses in SA. Doctors had the highest knowledge score followed by nurses and pharmacists, respectively. Practice scores did not corroborate knowledge and the higher attitude scores.

Self-assessment of knowledge showed that marginally more than half (< 55%) of HCPs were ≥ 75% confident in their knowledge on these topics. Confidence on prescribing antimicrobials showed that < 60% of doctors were confident in prescribing antimicrobials, with the confidence level even further reduced for pharmacists and nurses. Confidence in prescribing the correct treatment for given conditions ranged between 34% and 42% for the HCPs. Gaps in KAP were thus evident. Healthcare professionals indicated the need for more education and training, thus requiring a review of pre-service and in-service education and training in addition to CPD programmes for practising HCPs. It is recommended that both higher education institutions offering medical, pharmacy and nursing degrees as well as professional bodies regulating this education should map and update their existing curricula and scopes of practice, respectively, against the World Health Organization’s competency framework and associated curriculum for healthcare workers’ education and training on AMR.^22^

## Appendix 1

The knowledge, attitudes and practices of doctors, pharmacists and nurses on antimicrobials, antimicrobial resistance and antimicrobial stewardship in South Africa.

### Informed consent to participate in a research study

Dear Colleague,

We kindly request your participation in a national survey as a prescribing or dispensing health professional. The aim is to assess the knowledge, attitudes and practices (KAP) of doctors, pharmacists and nurses on antimicrobials, antimicrobial resistance (AMR) and antimicrobial stewardship (AMS) in South Africa (SA).

Antimicrobial resistance is a growing threat in both the developing and developed countries, causing problems in all spheres of human health, animal health, the food industry and agriculture.

**Antimicrobials** are substances of natural, semisynthetic or synthetic origin which kill or inhibit the growth of microorganisms whilst causing little to no harm or damage to the host. These include antibacterials, antivirals, antifungals and antiprotozoals.

**Antimicrobial resistance** occurs when the microorganisms withstand the antimicrobial agents which were initially effective in treating the infections caused by the microorganisms.

**Antimicrobial stewardship** is the coordinated programme which promotes the correct use of antimicrobials, improving patient outcomes, reducing AMR whilst decreasing the spread of infections caused by multidrug-resistant microorganisms.

The objectives of this study are as follows:

1. To assess the KAP of doctors, pharmacists and nurses on antimicrobials and AMR by means of a questionnaire survey.
2. To compare and contrast KAP within and between each profession.
3. To advise on education and training of these professions in terms of antimicrobials, AMR and AMS.

Your participation in this study involves answering questions about antimicrobials, AMR and AMS. The questions will be open-ended and close-ended and will take approximately 25 min. Your participation is entirely voluntary.

Please be advised that:

- No identifying information will be stored or disseminated. Be assured that answers are anonymous as all surveys will be aggregated first before analysis.
- Confidentiality and anonymity is guaranteed by the electronic survey format.
- Your participation is critical for the success of this research study, and entirely voluntary. You are free to decline from participation.
- There are no known risks in participating in this study.
- This research study has been granted ethical clearance from the Humanities and Social Sciences Research Ethics Committee of University of KwaZulu-Natal (Approval No HSS/0868/017M).
- If you wish to proceed and participate after reading this letter, you are giving voluntary informed consent by proceeding with the survey.

Mariette Snyman, Administrator of Humanities and Social Sciences Research Ethics Committee Office, may be contacted on +27(0)31 2608350 or snymanm@ukzn.ac.za.

Thanking you in advance for your participation. If you have any questions or queries, please feel free to contact the following:

**Researcher:** Reshma Balliram, Bachelor of Pharmacy on 9502731@stu.ukzn.ac.za.

**Supervisor:** Professor Sabiha Essack, South African Research Chair in Antibiotic Resistance & One Health on +27(0)31 2607785 or essacks@ukzn.ac.za.

We thank you in advance for your participation.

Yours faithfully,

Reshma Balliram

There are 42 questions in this survey.

### Section 1: Demographical information

**What is your gender?**

Please choose only one of the following:

**Table:**

Male ○	Female ○

**Which age group do you fall under?**

Please choose only one of the following:

**Table:**

20–25 years ○	36–40 years ○	51–55 years ○
26–30 years ○	41–45 years ○	56–60 years ○
31–35 years ○	46–50 years ○	60 years + ○

**What is your race group?**

Please choose only one of the following:

**Table:**

African ○
Indian ○
White ○
Coloured ○
Other ○

**In what year did you graduate?**

_____________

**At which University/College did you obtain your first degree or diploma?**

______________________________________________________

**What qualification did you obtain?**

**For doctors only:**

**Table:**

MBChB (Bachelor of Medicine and Bachelor of Surgery)	○
Master of Medicine	○
Doctorate (PhD in Medicine)	○
Specialist (Fellow of College)	○
Other	○

If other, specify ________________________________________

**For pharmacists only:**

**Table:**

Bachelor of Pharmacy	○
Master of Pharmacy	○
Doctorate (PhD in Pharmacy)	○
Other	○

If other, specify __________________________________________

**For nurses only:**

**Table:**

Diploma in Nursing	○
Bachelor in Nursing	○
Master of Nursing	○
Doctorate or PhD in Nursing	○
Other	○

If other, specify_________________________________________

**What is your field of practice?**

**Table:**

Public: Primary or community health centre	○
Public: District hospital	○
Public: Regional hospital	○
Public: Tertiary hospital	○
Private: Community	○
Private: Hospital	○
Academia	○
Other	○

If other, specify__________________________________________

If you are specialising, please state your area of specialisation and year obtained?

_____________________________________________________

**Where do you practice?**

**Table:**

Urban area	○
Peri-urban area	○
Rural area	○

**In which province do you practice?**

**Table:**

Gauteng	○	North West	○
KwaZulu-Natal	○	Eastern Cape	○
Limpopo	○	Free State	○
Mpumalanga	○	Northern Cape	○
Western Cape	○		

#### Section 2: Knowledge

**Would you consider antimicrobial resistance as a serious problem?**

**Please select your appropriate response for each item:**

**Table:**

	Strongly agree	Agree	Neutral	Disagree	Strongly disagree
Globally	○	○	○	○	○
In South Africa	○	○	○	○	○
In your hospital or practice	○	○	○	○	○

**How confident are you on your knowledge of antimicrobials, antimicrobial resistance and antimicrobial stewardship?** (Rate yourself on a scale of 1 to 5, with 1 being least confident and 5 being very confident)

**Table:**

	1	2	3	4	5
Antimicrobials	○	○	○	○	○
Antimicrobial resistance	○	○	○	○	○
Antimicrobial stewardship	○	○	○	○	○

**Are antimicrobial drugs effective in treating acute viral infections?**

**Table:**

Yes ○	No ○

**Common colds caused are caused by:**

**Table:**

Viruses ○
Bacteria ○

**Please select the factors you believe to be the most contributory towards antimicrobial resistance:**

○ Overusage of antimicrobials by prescriptions
○ Overusage of antimicrobials without prescriptions
○ Errors in medical prescriptions (dose, duration of use, choice)
○ Non-compliance of patients with prescribed treatment
○ Inadequate hand washing
○ Lack of immunisation campaigns
○ Lack of new antimicrobial drugs
○ Use of antimicrobials as growth promoters in animals
○ Patient pressure for antimicrobial prescriptions

**Which of the following sources do you refer to for information on appropriate usage of antimicrobials?** Please select your appropriate choice and rate on a scale of 1 to 5 (1 = lowest preference and 5 = highest preference):

**Table:**

	1	2	3	4	5
Information from senior experienced colleagues	○	○	○	○	○
South African Medicines Formulary	○	○	○	○	○
The Merck Manual	○	○	○	○	○
Medical journals	○	○	○	○	○
Medical websites	○	○	○	○	○
Standard Treatment Guidelines	○	○	○	○	○
International guidelines (e.g. Professional society guidelines)	○	○	○	○	○
	○	○	○	○	○

**Please assess your levels of confidence in the following areas when prescribing antimicrobials?**

(1 = very confident, 2 = confident, 3 = unsure, 4 = unconfident, 5 = very unconfident and 6 = not applicable)

**Table:**

	1	2	3	4	5	6
Making an accurate diagnosis of the infection	○	○	○	○	○	○
Decision not to prescribe antimicrobial when	○	○	○	○	○	○
patient has a fever with no serious criteria	○	○	○	○	○	○
and you are not sure of the diagnosis	○	○	○	○	○	○
Selecting the correct antimicrobial	○	○	○	○	○	○
Selecting the correct dosage for the antimicrobial	○	○	○	○	○	○
Selecting the correct interval for the antimicrobial	○	○	○	○	○	○
Selecting the correct duration for the antimicrobial	○	○	○	○	○	○
Using combination therapy if necessary	○	○	○	○	○	○
Interpreting microbiological laboratory results	○	○	○	○	○	○
When to stop or streamline the antimicrobial therapy	○	○	○	○	○	○
according to clinical evaluations and investigations	○	○	○	○	○	○
Selecting between an intravenous or oral antimicrobial	○	○	○	○	○	○

**Do you think there are risks associated with irrational use of antimicrobials?**

**Table:**

Yes ○	No ○

If you answered ‘Yes’, please list the risks.

______________________________________________________

**Do you think restricting antimicrobial usage is necessary to reduce antimicrobial resistance?**

**Table:**

Yes ○	No ○

Have you attended any workshops or had training on antimicrobials and antimicrobial stewardship?

**Table:**

Yes ○	No ○

If you selected ‘**Yes**’, kindly state when you attended the workshops or training.

______________________________________________________

**Would you like more education and training on antimicrobial use, antimicrobial resistance and antimicrobial stewardship?**

**Table:**

Yes ○	No ○

**Please list the three most resistant bacteria you are aware of** (with 1 being the most resistant and 3 being the least resistant):

1. ____________________________________________________
2. ____________________________________________________
3. ____________________________________________________

**What percentage of clinical antimicrobial use is unnecessary or inappropriate in South Africa?**

**Table:**

○	Between 1% and 20%
○	Between 21% and 40%
○	Between 41% and 60%
○	Between 61% and 80%
○	Between 81% and 100%

#### Section 3: Attitudes

**Antimicrobials are safe drugs that can be commonly prescribed?**

**Table:**

Yes ○	No ○

**Prescribing antimicrobials to a patient who does not really need them may ultimately have a negative impact on their health.** Please choose one option from the list:

**Table:**

Strongly agree	○
Agree	○
Neutral	○
Disagree	○
Strongly disagree	○

**In your opinion, do you think the problem of antimicrobial resistance is getting better or worse?**

**Table:**

Better ○	Worse ○

**What do you think are the important strategies to combat antimicrobial resistance?**

**Table:**

Select the most appropriate choices:	
Educational campaigns	○
Use of therapeutic guidelines	○
Vaccination campaigns	○
Improved infection control	○
Reducing antimicrobial use in agriculture and animals	○
Better control on antimicrobial sales	○
Other	○

If other, specify: ________________________________________

**Do you believe a patient skipping or missing one or two doses of antimicrobials contributes to the development of antimicrobial resistance?**

**Table:**

Yes ○	No ○

**Do you believe that new classes of antimicrobials will be developed in the next 5–10 years?**

**Table:**

Yes ○	No ○

**Do you believe that you may personally be contributing towards antimicrobial resistance?**

**Table:**

Yes ○	No ○

#### Section 4: Practice

**For pharmacists only: In addition to your Bachelor of Pharmacy degree, do you possess the Section 22A (15) permit allowing you to prescribe for primary care drug treatment?**

**Table:**

Yes ○	No ○

**For nurses only: Do you possess a concession permit in terms of Section 22A (12) from Medicines and Substances Control Act of 1965 and Section 38A of the Nursing act of 1978?**

**Table:**

Yes ○	No ○

**Do you prescribe antimicrobial drugs to your patients?**

**Table:**

Yes ○	No ○

Not applicable    ○

**Please select the average number of patients to whom you prescribe antimicrobials per day?**

**Table:**

Not applicable	○
Less than 5	○
Between 6 and 10	○
Between 11 and 20	○
Between 21 and 30	○
Between 31 and 40	○
Greater than 40	○

**Do you prescribe antimicrobials empirically (based on observation and experience)?**

**Table:**

Yes ○	No ○

Not applicable    ○

**Before initiating antimicrobial therapy, do you send samples for microbiology testing to inform the need for and/or choice of antimicrobial therapy?**

**Table:**

Yes ○	No ○

Not applicable    ○

**Do you possess the latest South African Standard Treatment Guidelines?**

**Table:**

Yes ○	No ○

**How often do you use the antimicrobial treatment guidelines when deciding upon antimicrobials for a patient?**

**Table:**

Always	○	Sometimes	○
Never	○	Not applicable	○

**Which three antimicrobials do you prescribe the most (if applicable)?**

1. ___________________________________________
2. ___________________________________________
3. ___________________________________________

**Have you changed your prescribing behaviour in light of antimicrobial resistance in the past 5 years?**

**Table:**

Yes ○	No ○

Not applicable    ○

If ‘Yes’, how? ___________________________________________

**What advice do you impart to patients on antimicrobial usage?**

○ The patient must complete the full course of antimicrobials for the recommended duration.
○ The patient must take the antimicrobials at the correct time intervals.
○ The patient should never share antimicrobial treatment with anyone.
○ The patient must not stop treatment even if they are feeling better after a few doses.
○ The patient must save the remaining antimicrobials for the next time they are unwell.
○ The patient must dispose of remaining antimicrobials appropriately.

#### Antimicrobial treatment

**For doctors only:**

**Please provide the required information in the table below on antimicrobial treatment.**

**Table:**

Condition	Laboratory culture (select appropriate column – Yes or No)		Choice of antimicrobial	Adjunct or other drug treatment
	Always – A	On treatment failure – B		
Acute pharyngitis				
Nasopharyngitis				
Acute otitis media				
Chronic otitis media				
Acute sinusitis				
Bronchitis				
Pneumonia				
Cystitis				
Pyelonephritis				

**For pharmacists only:**

**Please recommend which antimicrobial drugs you are authorised to prescribe for the following conditions (if applicable).**

**Table:**

	Recommended treatment
Acute diarrhoea in adults	
Chronic diarrhoea in adults	
Acne vulgaris of skin	
**Urinary tract infections:**	
Uncomplicated cystitis in adults	
Complicated cystitis in adults	
Complicated cystitis in pregnant women	
**Respiratory conditions:**	
Acute bronchitis in adults and adolescents	
Pneumonia uncomplicated (excludes paediatric and over 65 years)	
**Eye infections:**	
Bacterial eye infections, conjunctivitis (excludes newborn)	
**Ear, nose and throat conditions:**	
Otitis externa	
Otitis media acute	
Sinusitis, acute, bacterial	
Tonsillitis and pharyngitis	

**For nurses only:**

**Please recommend which antimicrobial drugs you are authorised to prescribe for the following conditions (if applicable).**

**Table:**

Condition:	Recommended treatment
Severe necrosing gingivitis	
Tick bite fever	
Acute meningitis	
Conjunctivitis	
Acute otitis media	
Acute sinusitis	
Bacterial tonsillitis	
Acute bronchitis	
Boil abscess	
Urinary tract infection, uncomplicated	

Thank you for your valuable time in participating in this study. Your response will be invaluable towards optimising rational antimicrobial use and AMS.

**Thank you for completing this survey.**

**Submit your survey.**
